# Consideration of genetic variation and evolutionary history in future conservation of Indian one-horned rhinoceros (*Rhinoceros unicornis*)

**DOI:** 10.1186/s12862-022-02045-2

**Published:** 2022-07-20

**Authors:** Tista Ghosh, Shrewshree Kumar, Kirtika Sharma, Parikshit Kakati, Amit Sharma, Samrat Mondol

**Affiliations:** 1 Animal Ecology and Conservation Biology Department, Wildlife Institute of India, Chandrabani, Dehradun, Uttarakhand 248001 India; 2 World Wide Fund for Nature-India, 172B Lodhi Estate, New Delhi, 110003 India

**Keywords:** Megaherbivore, Paleobiogeography events, Evolutionary significant units (ESUs), Rhinocerotidae family, Reintroduction program, Founder effect

## Abstract

**Background:**

The extant members of the Asian rhinos have experienced severe population and range declines since Pleistocene through a combination of natural and anthropogenic factors. The one-horned rhino is the only Asian species recovered from such conditions but most of the extant populations are reaching carrying capacity. India currently harbours ~ 83% of the global wild one-horned rhino populations distributed across seven protected areas. Recent assessments recommend reintroduction-based conservation approaches for the species, and implementation of such efforts would greatly benefit from detailed genetic assessments and evolutionary history of these populations. Using mitochondrial data, we investigated the phylogeography, divergence and demographic history of one-horned rhinos across its Indian range.

**Results:**

We report the first complete mitogenome from all the extant Indian wild one-horned rhino populations (n = 16 individuals). Further, we identified all polymorphic sites and assessed rhino phylogeography (2531 bp mtDNA, n = 111 individuals) across India. Results showed 30 haplotypes distributed as three distinct genetic clades (F_st_ value 0.68–1) corresponding to the states of Assam (n = 28 haplotypes), West Bengal and Uttar Pradesh (both monomorphic). The reintroduced population of Uttar Pradesh showed maternal signatures of Chitwan National Park, Nepal. Mitochondrial phylogenomics suggests one-horned rhino diverged from its recent common ancestors ~ 950 Kya and different populations (Assam, West Bengal and Uttar Pradesh/Nepal) coalesce at ~ 190–50 Kya, corroborating with the paleobiogeography history of the Indian subcontinent. Further, the demography analyses indicated historical decline in female effective population size ~ 300–200 Kya followed by increasing trends during ~ 110–60 Kya.

**Conclusion:**

The phylogeography and phylogenomic outcomes suggest recognition of three ‘Evolutionary Significant Units (ESUs)’ in Indian rhino. With ongoing genetic isolation of the current populations, future management efforts should focus on identifying genetically variable founder animals and consider periodic supplementation events while planning future rhino reintroduction programs in India. Such well-informed, multidisciplinary approach will be the only way to ensure evolutionary, ecological and demographic stability of the species across its range.

**Supplementary Information:**

The online version contains supplementary material available at 10.1186/s12862-022-02045-2.

## Background

The members of Rhinocerotidae family were once one of the most diverse and widely distributed terrestrial herbivores with complex evolutionary history [1]. By late Pleistocene, this family was reduced to only nine species (from more than 100 species) spread across Eurasia (seven species) and Africa (two species) [1, 2]. Subsequently, early Holocene global warming (after Last Glacial Maxima) triggered their extinction in western Eurasia and southward movement of eastern Eurasian rhinos, leading to their distribution across Southeast Asia [2, 3]. Further, the range of all Eurasian rhino species (Javan, Sumatran and One-horned rhino) were affected by a combination of natural and anthropogenic factors during Pleistocene-Holocene transition period [15–9 thousand years ago (Kya)] [1, 3–6], followed by recent events of exploitation of natural resources (during colonial era), industrialisation and poaching (since seventeenth century) [7–10]. Population size of the most widely distributed Javan rhinos (during Holocene) [11] were greatly reduced during human population expansion since 10,000 years ago [3], whereas the Sumatran rhino populations became fragmented and isolated (since Holocene) due to submerged Sundaland corridors (late Pleistocene) [6]. The one-horned rhinos faced climate-change driven habitat shrinkage in late Pleistocene [12]. Currently the Javan and Sumatran rhinos are categorized as Critically Endangered (~ 60 Javan rhino—[13] and < 100 Sumatran rhinos—[10]) and one-horned rhino as Vulnerable by IUCN (~ 3700 individual, [14]). Recovery of these species in their natural habitats requires deeper understanding of demography, ecology and genetics for appropriate conservation measures.

The one-horned rhino, being the only Asian species recovered from severe population decline in the past are critical for the evolutionary potential of this group. With a current population size of ~ 3700 individuals (increased from few hundred individuals in 1990s), it retains ~ 96% of the Asian rhino population [10, 13, 14]. As majority of the current one-horned rhino bearing areas in India and Nepal are reaching to their carrying capacities [15, 16], future conservation efforts are directing towards reintroduction-based programmes. Detailed genetic assessment of the existing rhino populations is critical in this regard since strong historical demographic declines has led to loss of genetic variation in all rhino species (Black rhino—[17], White rhino—[18], Sumatran rhino—[6], Javan rhino—[13]). For example, Liu et al. [1] suggested low population size and reduced genetic diversity across Rhinocerotidae family for an extended period of time. Similarly, mitogenome-based phylogeography reported low variation in both Sumatran [10] and Javan [13] rhinos, but no such data is available for one-horned rhinos.

In this paper, we investigated the phylogeography and evolutionary history of one-horned rhinos in India (henceforth Indian rhino) as it harbours 83% [19] of the global population of this species. We sequenced the polymorphic sites in the Indian rhino mitogenome in 111 wild individuals surveyed across seven extant populations covering the states of Assam, West Bengal and Uttar Pradesh. Further, we identified the Evolutionary Significant Units (ESUs) in Indian rhinos and suggested appropriate conservation measures to secure the evolutionary potential of this species. We believe that the results will provide the most exhaustive genetic information for Indian rhinos that would be useful in future reintroduction and population management efforts.

## Results

### Rhino mitogenome data and comparative analyses

Sequencing with 23 primers (Additional file 1: Table S1) generated 16,828 bp mitogenome (Additional file 2: Fig. S1) for wild Indian rhino (n = 16, Genbank: MZ736693–MZ736708, Additional file 1: Table S2). Comparison with the available one-horned rhino mitogenome data (Genbank: X97336) showed identical patterns of gene annotations. Composition analysis revealed AT-skewed mitogenome with 13 protein coding genes, 22 tRNA, 2 ribosomal genes and a non-coding control region (Additional file 1: Table S3). Comparative analyses with other rhino species (Additional file 1: Table S4 and Additional file 2: Fig. S2) revealed that the Indian rhinos have low segregating sites (S_Java_ = 15,514_,_ S_Africa_ = 10,680_,_ S_Sumatra_ = 130, S_India_ = 18) and nucleotide diversity (π_Java_ = 0.56_,_ π_Africa_ = 0.43_,_ π_Sumatra_ = 0.003, π_India_ = 0.0005) but high haplotype diversity (Hd_Sumatra_ = 0.96, Hd_India_ = 0.93, Hd_Java_ = 0.91_,_ Hd_Africa_ = 0.67). Both African rhino species (white and black rhino) data were combined for this analyses as no intra-species variation was observed in the available data.

### Phylogeography of wild Indian rhinos

**Fig. 1:**
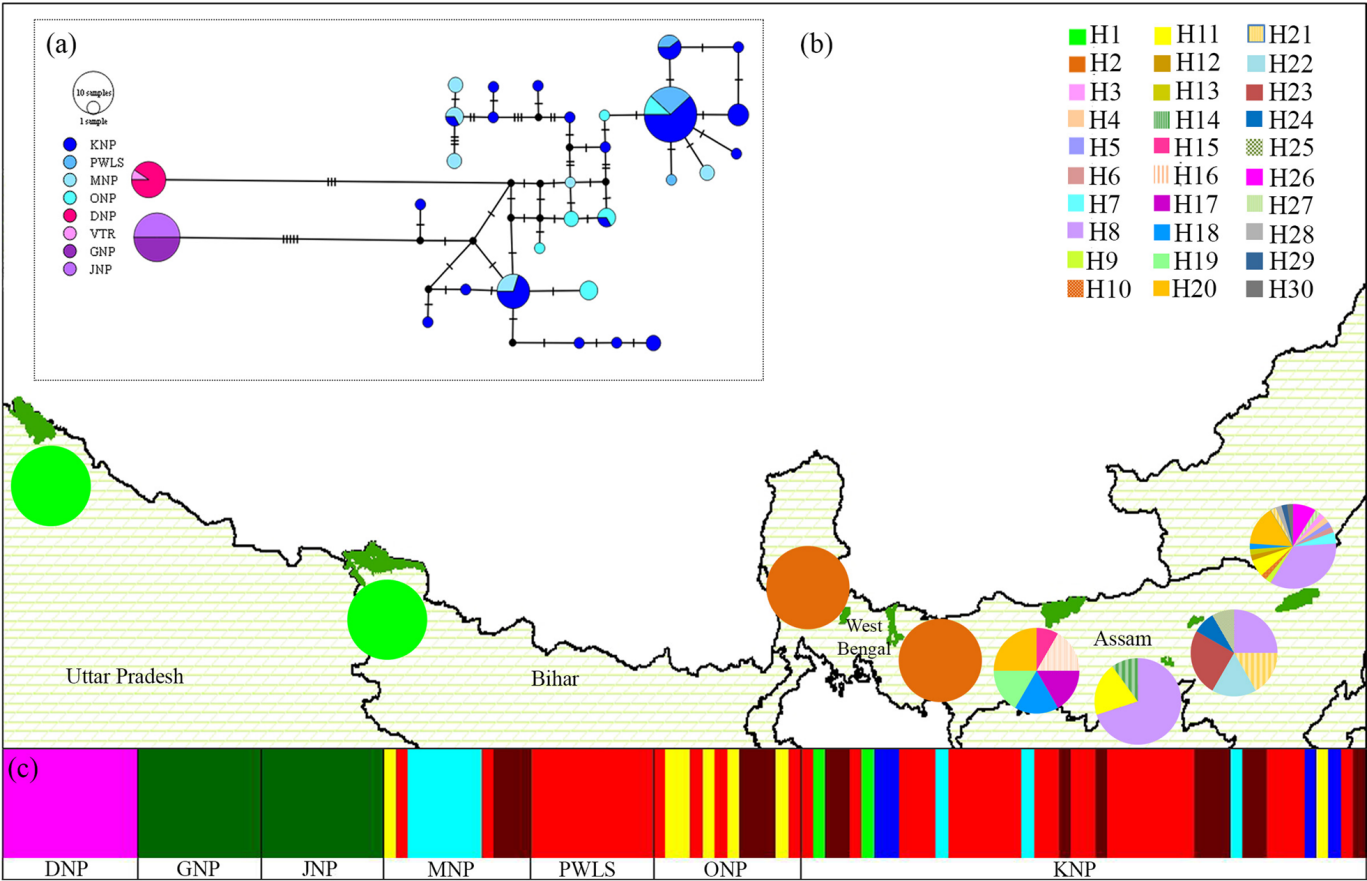
Representation of mtDNA variations and genetic structure in Indian rhinos based on 2531 bp concatenated sequence covering all polymorphic sites across seven genes. **a** Median joining network with park-level colour codes; **b** Haplotype frequencies at each of the sampled areas covering all variations (n = 30 haplotypes); **c** Bayesian clustering shows monomorphism in Uttar Pradesh (with sample from Bihar, n = 11) and West Bengal (n = 20) populations and polymorphism in Assam (n = 80)

Out of 15 primers designed to assess genetic variation, eight were finally used (Additional file 1: Table S1) to amplify all 21 polymorphic sites (covering 2531 bp sequence) of rhino mitogenome. This data was generated for additional 95 unique individuals (n = 56 tissue and 39 dung, Additional file 1: Table S2) (Genbank: MZ771364–MZ771458, MZ771459–MZ771553, MZ771554–MZ771648, MZ771649–MZ771743, MZ771744–MZ771838, MZ771839–MZ771933 and MZ771934–MZ772028). Remaining samples could not be used as they were rejected due to low amplification success for microsatellite data (n = 12), genetic recaptures (n = 13) and individuals from adjacent midden sites (n = 24). Sequencing comparison showed that out of the 21 polymorphic sites, two and three sites were specific to West Bengal and Uttar Pradesh, respectively, whereas all others were shared at different levels among the three states (shared among three states—10 sites, Assam–Uttar Pradesh—eight sites, Assam–West Bengal—four sites, West Bengal–Uttar Pradesh—0 sites, Additional file 1: Table S5). Median joining network (n = 111 individuals) showed a total of 30 haplotypes (h) across India. Majority of these haplotypes (93.3%, n = 28) were from Assam whereas both West Bengal (one haplotype, n = 20) and Uttar Pradesh (one haplotype, n = 10) populations were found to be monomorphic (Fig. 1). The sequence from Bihar rhino individual was identical to the Uttar Pradesh population. Population-wise genetic variation indices (Table 1) showed overall highest values for KNP (n = 46; S = 18, h = 19, π = 0.0021, Hd = 0.85), followed by MNP (n = 12; S = 14, h = 6, π = 0.0023, Hd = 0.89), ONP (n = 12; S = 9, h = 6, π = 0.0016, Hd = 0.89) and PWLS (10; S = 2, h = 3, π = 0.0002, Hd = 0.51). Bayesian genetic clustering corroborated with the earlier pattern (K = 3) where samples from West Bengal and Uttar Pradesh formed distinct clusters whereas Assam showed geographically intermixed fixed haplogroups (Fig. 1). The genetic differentiation (pairwise F_st_) values among these three clusters were significantly high ranging from 0.68 to 1 (Table 2, indicating highly structured populations). The hierarchical AMOVA analysis using two separate groupings: (a) seven populations and (b) three states showed higher within population (50%) and between group variance (45%) (Table 2). Such pattern indicates that overall genetic structure is influenced by differentiation at clade level and the amount of diversity present within the Assam clade (Tables 1 and 2).

**Table 1:** mtDNA diversity indices of all rhino populations in India (n = 111)

Protected areas	Sample size	Segregating sites (S)	Haplotypes (h)	Haplotype diversity (Hd)	Nucleotide diversity (π)
Kaziranga National Park, Assam	46	18	19	0.85	0.0021
Pobitora Wildlife Sanctuary, Assam	10	2	3	0.51	0.0002
Manas National Park, Assam	12	14	6	0.89	0.0023
Orang National Park, Assam	12	9	6	0.87	0.0016
Dudhwa National Park, Uttar Pradesh	10	0	1	0	0
Valmiki National Park, Bihar	1	NA	NA	NA	NA
Gorumara National Park, West Bengal	10	0	1	0	0
Jaldapara National Park, West Bengal	10	0	1	0	0
**Total**	**111**	**21**	**30**	**0.89**	**0.0028**

**Table 2:** Results of pairwise genetic differentiation and hierarchical AMOVA test (Bihar sample considered under Uttar Pradesh clade)

Pairwise F_st_ among clades (*p < 0.05)				
	Assam	Uttar Pradesh	West Bengal	
Assam	0			
Uttar Pradesh	0.68*	0		
West Bengal	0.73*	1.0*	0	

AMOVA test among three clades and seven populations				
Source of variation	d.f	Sum of squares	Fixation index	Percentage of variation
Among groups	2	14.689	0.45 (F_ct_)	44.66
Among populations within groups	4	3.233	0.1 (F_sc_)	5.71
Within populations	104	31.267	0.50 (F_st_)	49.63
Total	110	49.189	0.60573	

### Divergence time of different rhino clades and demographic history

**Fig. 2:**
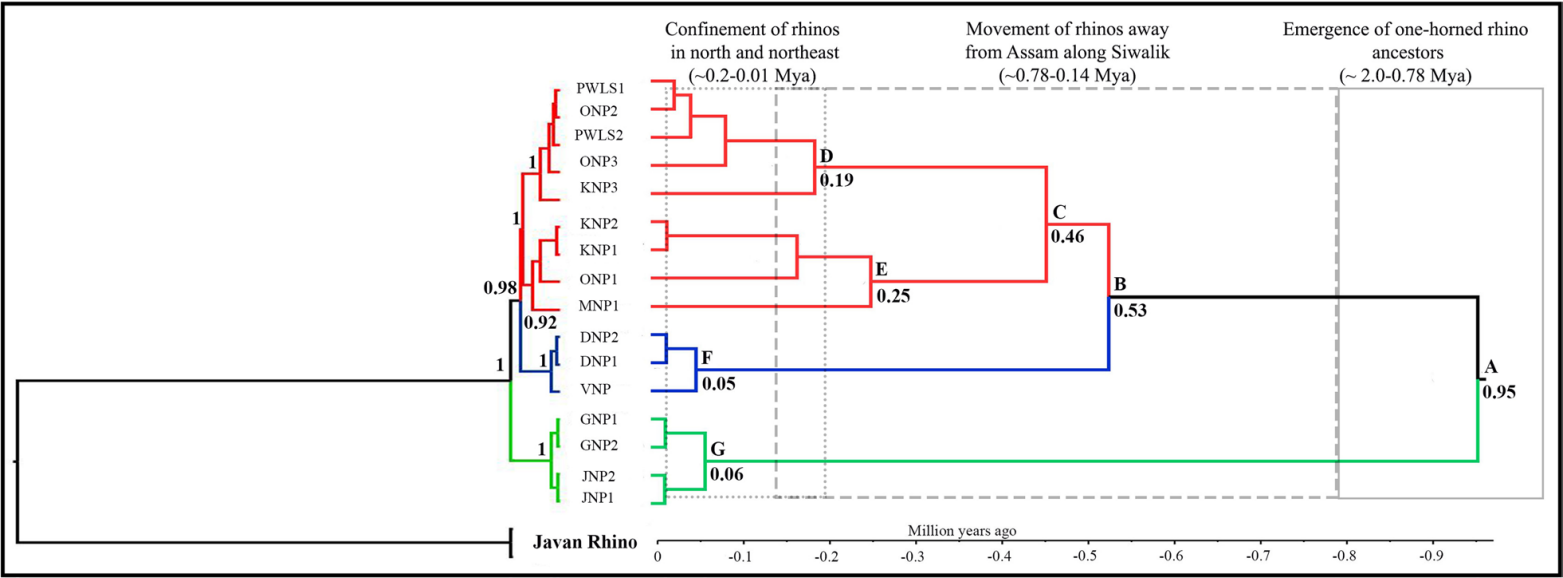
Phylogenetic relationship and assessment of divergence time in Indian rhino populations. The left pane shows the clustering of three maternal clades of West Bengal samples (green), Uttar Pradesh (blue) and Assam (red). Javan rhino sequence was used as outgroup. The posterior probability values (≥ 0.9) are shown in bold. The right pane indicates the divergence of Indian rhinos ~ 0.95 Mya, where the Assam population coalesce first (~ 0.19 Mya), followed by divergence of West Bengal and Uttar Pradesh (0.06–0.05 Mya). Node-specific ages are marked (with posterior probability values ≥ 0.9). The major corroborating paleobiogeographical events are presented above

The Bayesian phylogeny showed similar pattern of three clades consisting of West Bengal, Assam (nodes C–E) and Uttar Pradesh (along with the Bihar sample, Fig. 2). Based on the calibrated root nodes and Indian rhino-specific mutation rate (1.2 × 10^–4^ mean rate of substitution per site per million years, Additional file 2: Fig. S3), tMRCA analysis suggested a divergence period spanning from 950 (HPD 1360–810 Kya) to 50 (150–10 Kya) Kya (Fig. 2). Our results indicated the divergence of Indian rhinos ~ 950 Kya (node A, Fig. 2) corresponds to the emergence period of one-horned rhino ancestors in the subcontinent [5, 12]. Next, the Assam population diverged from the remaining clades at ~ 500 Kya (HPD 680–330 Kya, nodes B & C, Fig. 2). This is supported by reports of multiple rhino movements away from Assam (along Siwalik as well to Siva-Malayan region) during this period [12, 20]. At population level, results suggest a relatively earlier coalescence of Assam ~ 190 Kya (HPD 300–70 Kya, node D & E, Fig. 2) compared to West Bengal and Uttar Pradesh (~ 50 Kya, HPD 150–10 Mya, node F & G, Fig. 2). This period (120–10 Kya) is known for confinement of rhinoceros to the north and north-east of India due to monsoon intensification and grassland dominance [5, 12, 21].

**Fig. 3:**
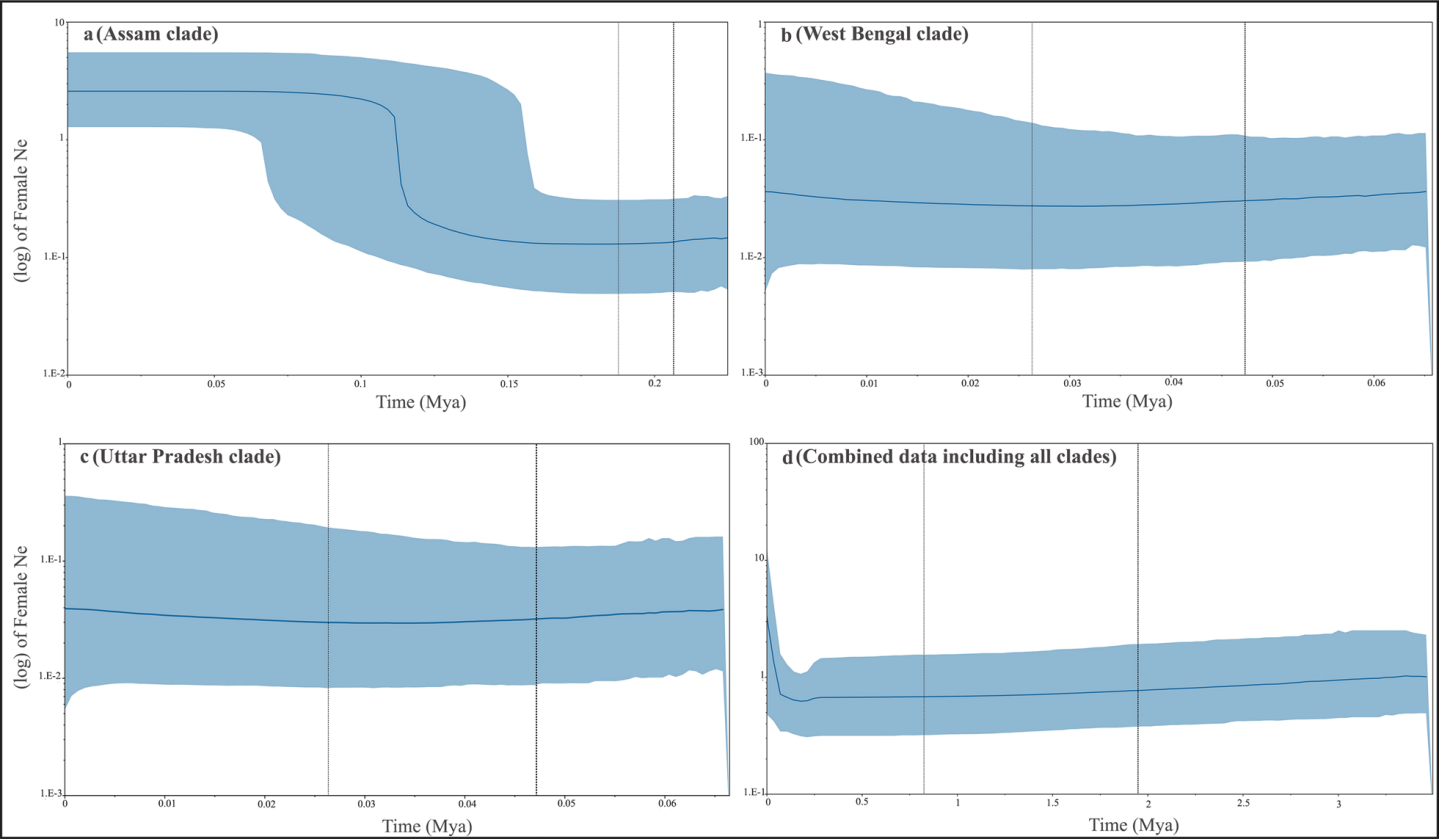
Bayesian skyline plot analysis (BSP) to determine the changes in female effective population size across three clades, **a** Assam, **b** West Bengal, **c** Uttar Pradesh and **d** combined dataset of Indian rhinos. The vertical lines represent the HPD intervals of the given divergence time for each analysis whereas the shaded horizontal area is the HPD of the median effective size value

All four BSP analyses showed similar population trends for overlapping time periods where the combined data identified trends at a deeper coalescence period compared to the clade-specific data (Fig. 3). The Assam clade showed a steep increase in female effective population size ~ 110 Kya followed by constant population size from ~ 90 Kya (Fig. 3a) whereas West Bengal and Uttar Pradesh clades showed similar demographic trends of stable populations from ~ 60 Kya (Fig. 3b, c). The combined dataset showed a steep decline in population size ~ 300–200 Kya followed by a gradual increase ~ 120 Kya and steep rise ~ 60 Kya (Fig. 3d).

## Discussion

This study presents the most extensive mitochondrial DNA phylogeography of one-horned rhinos across its Indian distribution. Careful considerations involving mitogenome sequencing of representative samples across Indian rhino-bearing areas, identification of all polymorphic regions and their amplification from spatially-covered rhino samples helped us achieving accurate assessment of mtDNA variations. To the best of our knowledge, this is the first report of wild Indian one-horned rhino mitogenome from all the extant populations. Despite relatively similar haplotype diversity of Asian rhinos [India—0.93 (16 samples), Sumatra—0.96 (15 samples), and Java—0.91 (6 samples), respectively], Indian rhino mitogenome showed much lower values for segregating sites and nucleotide diversity (Additional file 1: Table S4). Such mitogenome comparisons may be affected by limited sample size (earlier studies in African rhinos have reported higher diversity based on partial mitogenome data with more samples [17, 18]) or representation of historical genetic variations (in Javan rhinos, [13]). However, it was surprising to observe that despite similar historical demographic incidences (severe population decline due to habitat shrinkage [6, 12] and anthropogenic pressures [9, 10]) Indian rhino retain much lower genetic variation than their Sumatran counterpart. This can be potentially attributed to recovery of the Indian species from extremely low founder population (as indicated by high Hd but low π) [22, 23].

As expected, the phylogeography data (2531 bp mtDNA, n = 16 samples) revealed higher number of haplotypes than the mitogenome data (n = 30 haplotypes) (due to large sample size). The only other study on one-horned rhino mtDNA variations (based on partial control region sequences, 428 bp) reported 10 haplotypes (Kaziranga National Park, India—4 and Chitwan National Park, Nepal—6, respectively) and moderate level of genetic difference (F_st_ value of 0.39 between them) [24]. Careful scrutiny of our data revealed that all the polymorphic sites (or identified segregating sites) were found in fixed positions within one-horned rhino mitogenome (Additional file 1: Table S5) across India. Given the distribution of polymorphic sites in the sequenced mitogenomes and our sampling coverage, it is likely that these data represent the majority or perhaps all extant mtDNA haplotypes in Indian rhinoceros populations. This claim is also supported by the similar haplotype diversity values from the mitogenome and the phylogeography datasets (0.93 and 0.9, respectively). Our study also shows that the Indian rhinos have the highest number of haplotypes compared to the other genus/species reported so far [10, 13, 17, 18]. The clustering analysis of the concatenated rhino sequences showed three distinct genetic clades (corresponding to the states of Assam, West Bengal and Uttar Pradesh) with high F_st_ value (0.68–1), corroborating with the haplotype network patterns. Mantel test (− 0.83, p = 1) confirmed that such strong genetic structuring is not due to isolation by distance pattern, but driven by lineage-specific evolutionary history (as suggested by AMOVA results). Such pattern of higher within population and between group variance (50% and 45% in Indian rhinos, respectively) indicates that the mitochondrial genetic variation observed in extant Indian rhino is influenced by both evolutionary diversification and retention of diversity at population level only for Assam clade. As two of the clades are monomorphic, they contribute very less proportion of among-population within-group variations (5%). Similar data has also been described in other species such as barking deer—[25], dog—[26] etc. Interestingly, we found that the sequence from the Bihar sample (representing samples from Nepal) was identical to the Uttar Pradesh sequences, including the state-specific SNPs. This pattern was expected as the founder animals of the reintroduced Uttar Pradesh population were sourced from Chitwan National Park, Nepal (four dominant breeding females) and Pobitora Wildlife Sanctuary of Assam (dominant breeding male) [27]. Further comparison of 13 partial D-loop sequences from Chitwan National Park, Nepal Zschokke et al. [24] confirmed this pattern, indicating that the mtDNA signature of the Uttar Pradesh population belongs to Nepal. Given that the entire Uttar Pradesh rhino population showed only one haplotype, future studies need to evaluate the mtDNA variation in the Nepal population.

The phylogenetic analyses reconfirmed the relationship among the existing members of the Rhinocerotidae family [10, 28, 29] where the Sumatran and African rhino formed sister clades, separated from the *Rhinoceros* sp. based on the extant rhino genus/species sequence data only (Woolly rhinoceros sequence was not used). The within species tree topology corroborated with the haplotype network results as Assam and Uttar Pradesh formed phylogenetically closer clades as compared to West Bengal. We believe that the observed phylogenetic pattern of West Bengal being separate clade is influenced by lesser shared polymorphic sites between West Bengal and other two clades (Additional file 1: Table S5). Combined together, we interpret that the one-horned rhino diverged from its recent common ancestors ~ 950 Kya and different populations (Assam, West Bengal and Uttar Pradesh/Nepal) coalesce around ~ 190–50 Kya time period (Fig. 2). The molecular dates were comparable to other published literature on rhino evolution [5, 12, 21] and supported by the paleobiogeographic history of the Indian subcontinent [12, 21]. For instance, the inward movement of rhinos from Assam along Siwalik (680–330 Kya, node B & C) coincides with drop in the sea level which facilitated movement of multiple genera (for example, *Elaphas, Panthera*, *Rhinoceros*, *Muntiacus* etc.) through Siva-Malayan route [20, 30]. Report of one-horned and Javan rhino co-existence in Bhutan ~ 560 Kya [13] provide further support of such movements. Finally, the coalescence time of the three Indian clades (~ 190–50 Kya, Fig. 2) corresponds to Holocene climatic optimum period known for monsoon intensification in north and north-east part of India resulting in range contraction for grassland dependent species [5, 12, 31]. We feel that our approach of using taxon-specific mutation rate and fossil data for node calibration has resulted in achieving such meaningful estimates of tMRCA. Future efforts should try to include molecular data from historical/ancient samples to tighten the variance associated with divergence estimates [32]. Overall, this approach reiterates the critical importance of large datasets (whole mitogenome from multiple individuals in this case), informative prior settings and its assessment with posterior outputs, taxon-specific mutation rate, node calibration points etc. for accurate tMRCA estimation [33–37].

The BSP results with different datasets (combined vs. three individual clades) showed similar patterns of changes at different evolutionary timescale. The combined data indicate a historical decline in maternal effective population size ~ 300–200 Kya, followed by increasing tends during ~ 110–60 Kya (coinciding with Holocene climatic optimum period, also seen in the Assam clade analysis, Fig. 3). This pattern is similar to earlier findings (described in [1] based on whole genome data) with a difference in the Ne values arising from lower effective size in mtDNA [38]. The West Bengal and the Uttar Pradesh clades did not show any changes in population trajectories owing to the monomorphic data. It is noteworthy to point out that such mitochondrial DNA-based analyses would only capture the demographic history at longer evolutionary time scale, and use of suitable nuclear markers (microsatellites, SNPs etc.) could provide much powerful demographic inferences [38].

The spatially exhaustive sampling coverage and the patterns of population structure brings out some critical conservation perspectives for the Indian rhinos. The phylogeographic and mitophylogenomic patterns suggest three distinct clades with state-specific evolutionary histories. As these populations are morphologically undistinguishable and interbreed among themselves (Dudhwa, Uttar Pradesh population is genetically mixed, [27]), we suggest that they should be recognised as ‘Evolutionary Significant Units (ESUs)’ [39]. It is therefore important to use such information towards conservation and management efforts for each of these populations [39–41]. Despite strong recoveries across all existing populations since late 1990s, recent analyses suggest high extinction probability of the species [42], and further conservation efforts are mostly concentrated on translocation activities [16, 43]. Till date genetic information of the species has not been used in translocation planning (possibly due to lack of sufficient data), and the genetic signatures described in this study would be very helpful to increase variation in target populations. For example, the Uttar Pradesh and West Bengal population show state-specific monomorphic haplotypes representing unique but genetically depauperate populations. Based on the data presented here, suitable founder animals from Assam populations can be considered for future translocation programs in these areas, thereby increase the genetic diversity of these populations to combat any potential stochastic events [40, 41]. However, such efforts would impact the suggested ESU categorizations due to mixing of different gene pools among populations. Another important aspect for management consideration would be better planning for translocation events to any of the existing or new areas [16, 43]. For example, the reintroduced rhino population in Assam (Manas National Park) showed much higher mtDNA variation (six haplotypes), possibly due to periodic supplementation of individuals of varied genetic ancestry across different wild rhino populations [44] compared to Dudhwa NP (single haplotype) of Uttar Pradesh (single supplementation event). As multiple reintroduction programs are planned as per the ‘National Conservation Strategy for the Indian One horned rhinoceros (*Rhinoceros unicornis*), Government of India, Ministry of Environment Forest and Climate Change, 2021’ objectives (in the states of Uttar Pradesh, Bihar, West Bengal and Assam) in near future, we suggest that all future efforts should adopt the Manas NP model with consideration of selecting genetically variable founder animals, multiple reintroduction events etc. [16, 45].

## Conclusion

The one-horned rhino was found throughout the Indo-Gangetic plains during the early twentieth century [43, 46] but faced drastic reductions in distribution and population size (including local extinctions) [47, 48], followed by one of the most successful species recovery (increase in population size) in wild across the world [7, 43, 48]. We present the first assessment of range-wide mitogenome diversity in Indian rhinos where we emphasize the importance of large data, spatial sampling coverage of populations and evolutionary history as fundamental information for future population reintroduction/recovery programs (as suggested in case of other species [49–52]). Our results are important for Indian rhino conservation because they suggest higher genetic diversity than earlier reported [24]. However, the existing habitats are small, disjunct, isolated and reaching their respective carrying capacities [16, 48] and conservation options are becoming limited except establishing new habitats and translocation-driven population enhancement [16]. We believe that the genetic information provided here will assist in identifying appropriate source populations and maintain adequate genetic diversity in the existing (and new) rhino populations, thereby ensuring evolutionary, ecological and demographic stability for their future survival.

## Methods

### Permission and ethical considerations

Data generated in this study is part of a collaborative programme titled “Implementing Rhino DNA Indexing System to counter rhino poaching threat and aid population management in India” (henceforth RhoDIS-India). Biological sampling from all the three rhino bearing states was permitted by Ministry of Environment, Forests and Climate Change (MoEF&CC), Government of India (Letter No. 4-22/2015/WL). Permission for dung sampling was provided by state forest departments of Assam (Letter No. A/GWL/RhoDIS/2017/913, 3653/WL/2W-525/2018, WL/FE.15/22), West Bengal (Letter No. 3967/WI/2W-525/2018) and Uttar Pradesh [Letter No. 1978/23-2-12 (G)]. We have also received one tissue sample from Valmiki National Park (henceforth NP), Bihar forest department assumed to be representing the wild rhinos of Nepal (Letter/no.-1296 dated 16.10.2020). No ethical permissions were required for tissues as they were collected from naturally dead rhinos as well as for dung samples.

### Study area

**Fig. 4:**
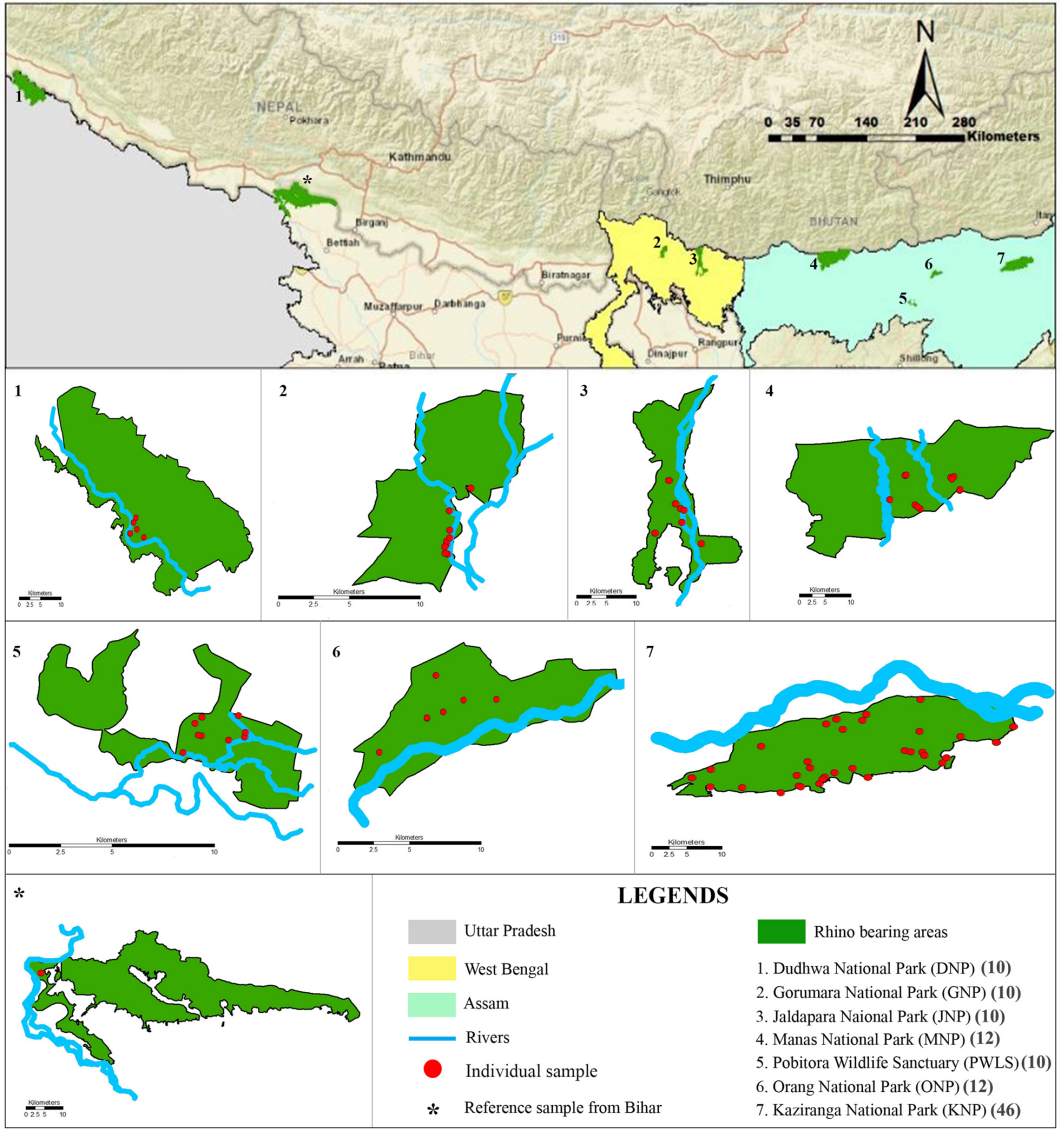
Map of the study area and distribution of the final samples used in this study (n = 111). The top plate shows the position of the rhino-bearing parks across three Indian states (Uttar Pradesh, West Bengal and Assam). The reference sample of wild rhino received from the state of Bihar (*) is also presented

During the 1600s, the one-horned rhinos were distributed throughout the northern Indian subcontinent covering all the major river basins from Pakistan to Indo-Myanmar borders. The species has lost most of its habitat and population size due to a range of anthropogenic interventions (habitat loss, hunting, poaching etc.) [7, 9, 14] and are currently distributed across only 12 protected areas covering > 2000 km^2^ area in India and Nepal [14]. This study on the Indian rhino was conducted across all extant rhino-bearing parks (n = 7) found across the states of Assam (n = 4 parks), West Bengal (n = 2 parks) and Uttar Pradesh (n = 1 park) situated in Terai-Duars region of north and north-east India (Fig. 4). Assam currently hosts the largest population of Indian rhinos (~ 80% of the total population), which are found across four isolated parks: Kaziranga NP, Orang NP, Pobitora Wildlife Sanctuary (WLS) and Manas NP. All of these populations have experienced severe hunting and poaching pressures during early-late 1990s, leading to local population sizes ranging from 50-few hundreds and local extinction in Manas NP, but have revived to their current population sizes [7, 14]. Between 2010 and 2020 Manas received 35 translocated rhinos from Kaziranga NP and Pobitora WLS part of the population recovery program [44]. The most recent population estimates of these parks are as follows: Kaziranga NP: ~ 2500; Orang NP: 100; Pobitora WLS: 101; and Manas NP: 42 [14].

The state of West Bengal currently retains ~ 350 rhino individuals distributed between two parks, Gorumara NP (52 individuals) and Jaldapara NP (> 250 individuals). This population has recovered from a severe population decline of ~ 20 individuals during early 1900s (due to severe habitat loss) [9]. The rhino population in Uttar Pradesh was locally extinct along with the entire Terai in mid 1990s (mostly due to habitat loss and hunting). During 1984–85, rhinos were reintroduced in Dudhwa NP (Uttar Pradesh) from Chitwan NP, Nepal and Pobitora WLS, Assam and currently this park hosts ~ 40 rhinos [27]. Apart from this, wild rhinos are occasionally reported from Valmiki NP, Bihar (adjacent to Chitwan NP, Nepal in the Indian part of Terai). These rhinos are either swept down by natural flooding or use the grasslands along the river Gandak within Valmiki NP during monsoon seasons.

### Biological sampling

Overall the sampling strategy in this phylogeography study was to select unique rhino individuals from different parts of the species distribution in India. A total of 160 samples (72 tissues and 88 dung) covering four states (Fig. 4) were used to assess rhino mitochondrial genetic diversity. The tissue samples of naturally dead rhino were provided by respective forest departments as part of RhoDIS-India protocol (2017–2021). Further dung collection was done to ensure spatial coverage for areas with no representative tissue samples. Rhino dung sampling can be challenging in the wild due to their use of communal latrine system (middens) [53, 54]. In this study, sampling was conducted by intensive foot and vehicle surveys from already known midden sites across six rhino bearing parks (except Kaziranga NP). During sampling, only the fresh bolus from top of the midden was selected and swabbed twice with separate PBS-soaked sterile cotton swabs (Himedia, Mumbai, India). All samples were geo-tagged and transferred to laboratory in − 20 °C freezer till downstream processing.

### DNA extraction

Tissue DNA was extracted using already established protocol for Indian rhino mentioned in Ghosh et al. [55]. For dung samples, a modified protocol from Biswas et al. [56] was used. In brief, samples were digested overnight with a combination of 700 μl ATL and 65 μl Proteinase K (20 mg/ml) at 56 °C, followed by QIAamp DNA Tissue Kit (QIAGEN Inc., Hilden, Germany) protocol with adjusted volumes. DNA was eluted twice in 100 μl preheated (70 °C) 1X TE buffer and stored in − 20 °C freezer. Extraction negative was used for each set of extraction (n = 23) to monitor possible contamination.

### PCR amplification and sequencing

To assess genetic variation of the extant rhino populations, complete mitogenome data was generated for representative samples from each park (n = 15, see Additional file 1: Table S2 for details) and one from the Valmiki National Park, Bihar. These samples were selected based on their geographic locations representing the farthest samples within each park to ensure inclusion of potentially unrelated individuals. Mitogenome sequencing was performed using already published 23 overlapping primers [57]. For annealing temperature standardisation, gradient PCR was set in 10 μl reactions containing 4 μl of 2× Qiagen PCR buffer mix (QIAGEN Inc., Hilden, Germany), 1 μl of primer (3 μM), 2 μM BSA (4 mg/ml), 1.4 μl of RNase free water and 5 ng of rhino tissue DNA. PCR conditions included an initial denaturation (95 °C for 15 min); 35 cycles of denaturation (95 °C for 30 s), annealing (50–60 °C gradients for 40 s) and extension (72 °C for 40 s); followed by a final extension (72 °C for 10 min). During each set of reactions, PCR and extraction negatives were included to monitor contamination. Amplified products were visualized with 2% agarose gel, cleaned with Exonuclease (Thermo Scientific, Waltham, USA) and Shrimp Alkaline Phosphatase (Amresco, Solon, USA) mixture and sequenced bidirectionally in an ABI 3500XL bioanalyzer (Applied Biosystems). Out of these 23 primers, two did not show amplification in any samples. The remaining sequences (n = 21 from 16 individuals) were aligned with the available one-horned rhino mitogenome (Genbank: X97336, [28]) in Mega v7 [58]. Two primers were designed manually in the flanking conserved regions adjacent to the gaps (Additional file 1: Table S1) and sequences were generated from all the samples (n = 16).

The complete mitochondrial sequences (n = 16) were aligned and manually screened to identify the segregating sites. Further, a total of 15 primers were designed (multiple primers covering the segregating sites) to amplify all the polymorphic sites as < 500 bp fragments to ensure higher success rate from—poor quality dung DNA samples. These primers were standardised following same protocol described above. For all field collected samples (tissue = 56 and dung = 88) individual identification was performed using a panel of 14 microsatellites (described in [55]). After PCR amplification and genotyping of the markers, samples with 12–14 loci data were selected for downstream analysis and genetic recaptures were removed. To ensure removal of closely related individuals in our dataset we selected one sample from adjacent midden sites. Sequence data (2531 bp covering seven genes) was generated for the selected individuals to assess phylogeography patterns.

### Complete mitogenome annotation and comparative analysis

All rhino sequences (n = 16) were aligned in Mega v7 to generate a complete mitogenome sequence and manually checked to identify any nucleotide ambiguities. Annotation was done using MITOS2 web with default settings and vertebrate mitochondrial genetic code [59] followed by mitogenome map construction with OGDRAW [60]. The mitogenome annotation was further confirmed with earlier published one-horned rhino mitogenome data (Genbank: X97336, [28]). To ascertain species-wise mitochondrial DNA diversity these sequences were aligned with already published rhino mitogenome sequences from *Diceros bicornis* (n = 2, Genbank: FJ905814, NC012682 [61]), *Ceratotherium simum* (n = 2, Genbank: Y07726, NC001808 [62]), *Dicerorhinus sumatrensis* (n = 15, Genbank: MF066629-MFO66643 [10] and *Rhinoceros sondaicus* (n = 6, Genbank: FJ905815 [61], MK909142, MK909146, MK909148, MK909149, MK909151 [13]). We calculated number of segregating sites (S), nucleotide (π) and haplotype diversity (Hd) using DnaSP v.5 [63] for all genes in the mitogenome.

### Genetic diversity in Indian rhinos

Population-wise basic indices of genetic variations (S, π and Hd) were calculated for concatenated sequence data (2531 bp from seven genes) using DnaSP v.5 followed by a median joining [64] haplotype network constructed in PopART v. 1.7 [65]. To ascertain any possible population structure a Bayesian approach implemented in BAPS v.5.3 was used as it considers linked loci data [66]. Pairwise F_st_ and differential hierarchical AMOVA analysis was performed using Arlequin v. 3.0 [67] to confirm the pattern found in BAPS analysis.

### Estimation of clade-specific divergence times and demographic history

To identify the clades, Bayesian phylogeny was constructed with MrBayes v. 3.2.7 [68] using 16 Indian rhino mitogenome and Javan rhino sequence (outgroup, as they are the sister clade of one-horned rhinoceros) [69]. Analysis was conducted using GTR + G substitution model determined by jModelTest v2.1.3 [70] (based on Akaike Information Criteria). The MCMC parameters included 2 runs of four chains each of 15 million generations with sampling after 1000 generations till split frequencies were below 0.01. Posterior probabilities were calculated for each node.

To estimate divergence among clades, rate of mutation for Indian rhino was calculated using BEAST v.2.3.6 [32]. Analysis was performed with five extant rhino mitogenome (without D-loop) (n = 11 sequences, India = 7 (haplotypes representing maximum variation in the data), Java = 1, Sumatra = 1, White = 1, Black = 1) along with horse (*Equus caballus*, Genbank: NC001640), donkey (*Equus asinus*, Genbank: NC001788), Asiatic wild ass (*Equus hemionus*, Genbank: NC016061) and zebra (*Equus zebra*, Genbank: NC018780) as outgroups. GTR + G substitution model was selected through jModelTest v2.1.3 for this multi-species data. Birth–death speciation was considered as tree prior [10, 34] along with uncorrelated relaxed log normal clock [10, 33]. During analysis, four established internal node calibration points (based on fossil records) with normal distribution priors were employed: (i) Caballine split (4 ± 0.5 million years ago (Mya)) [71, 72]; (ii) late Oligocene diversification of rhino groups (26 ± 3.5 Mya) [73]; (iii) split of rhinoceros genus (3 ± 0.5 Mya) [1]; (iv) origin of the perissodactyls (55 ± 3 Mya) [74, 75]. The first three calibration points were considered as monophyletic constraint [33] as the last point includes both ingroup and outgroup taxa.

tMRCA (time to Most Recent Common Ancestor) was inferred using the estimated mutation rate with lognormal distribution under strict molecular clock (intra species data, n = 16) [34, 35]. MCMC runs included 100 million generations, sampled at every 10,000 states with 10% burn-in. Data convergence was checked with Tracer v. 1.5 [76] and the final tree (with maximum clade credibility) was estimated with TreeAnnotator [77] and visualised using FigTree v.1.4.2 [78].

To estimate past fluctuations in population size, Bayesian skyline analysis (Bayesian skyline plot or BSP) was conducted using concatenated sequence data with monophyletic constraint to the identified maternal clades ensuring phylogenetic construction. Analysis was conducted with multiple datasets (each clade and combined data, respectively) to ascertain any possible impacts of genetic structure in the data [38]. In all cases coalescent BSP tree prior was used along with strict molecular clock, estimated mutation rate and clade specific divergence date. MCMC parameter settings and data convergence were identical to the tMRCA analysis.

## Supplementary Information

**Additional file 1: Table S1.** Details of the primers used in sequencing Indian rhino whole mitogenome and phylogeography data. **Table S2.** Sample (both tissue and dung) of Indian rhinos used in this study. A total of 111 individual rhino samples (72 tissue and 39 dung samples, respectively) were used in this study. Out of the 72 tissue samples, 16 samples representing different parks were used to generate the whole mitogenome data. **Table S3.** Mitogenome organization in *Rhinoceros unicornis*. Codons respective to each tRNA are mentioned in parenthesis. **Table S4.** Comparative analysis results of genetic diversity indices among five extant rhino species. **Table S5.** Details of the variable sites based on concatenated sequence of 2531bp of Indian one horned rhino mtDNA haplotypes.

**Additional file 2: Figure S1. **Whole mitogenome organisation and annotation of *Rhinoceros unicornis*. **Figure S2.** Gene-wise comparative analyses of polymorphism indices (S, Hd and π) for all extant rhino species. **Figure S3.** Estimation of mitogenome mutation rate for Indian rhino using Caballine (Zebra, Donkey, Asiatic Wild Ass and Horse) as outgroup. Internal node calibration points are presented in italics (bold). Estimated node ages and branch mutation rates with available reference in literature is marked with * [1, 8, 10, 29].

## Data Availability

The dataset generated in this study is available in GenBank with Accession numbers MZ736693–MZ736708 (whole mitogenome sequences) and MZ771364–MZ771458, MZ771459–MZ771553, MZ771554–MZ771648, MZ771649–MZ771743, MZ771744–MZ771838, MZ771839–MZ771933 and MZ771934–MZ772028 (fragmented sequence data for phylogeography). The additional figures and tables are provided as Additional file 1: Tables S1–S4 and Additional file 2: Figs. S1–S3).

## Abbreviations

BSP: Bayesian skyline plot
ESUs: Evolutionary significant units
Kya: Thousand years ago
mtDNA: Mitochondrial DNA
Mya: Million years ago
Ne: Effective population size
NP: National Park
tMRCA: Time to the most common recent ancestor
