# Supplementary material for: Consideration of genetic variation and evolutionary history in future conservation of Indian one-horned rhinoceros (Rhinoceros unicornis)
Source: BMC Ecol Evol. 2022 Jul 20;22:92. doi: 10.1186/s12862-022-02045-2 (PMC9301832; doi:10.1186/s12862-022-02045-2)
Supplement: Supplementary file 1 — Additional file 1: Table S1. Details of the primers used in sequencing Indian rhino whole mitogenome and phylogeography data. Table S2. Sample (both tissue and dung) of Indian rhinos used in this study. A total of 111 individual rhino samples (72 tissue and 39 dung samples, respectively) were used in this study. Out of the 72 tissue samples, 16 samples representing different parks were used to generate the whole mitogenome data. Table S3. Mitogenome organization in Rhinoceros unicornis. Codons respective to each tRNA are mentioned in parenthesis. Table S4. Comparative analysis results of genetic diversity indices among five extant rhino species. Table S5. Details of the variable sites based on concatenated sequence of 2531bp of Indian one horned rhino mtDNA haplotypes. [file 12862_2022_2045_MOESM1_ESM.pdf]

Additional file 1 Table S1: Details of the primers used in sequencing Indian rhino whole mitogenome and phylogeography data.

S.No.	Primer	Sequence	Ta (°C)	Amplicon	Targeted genes	Polymorphic sites	Genbank accessions
1	P2[*]	F: CACTGAAAATGCCTAGATGAG<br>R: CTAGGTGTAAACTAGRTGCTT	55	~950	$tRNA^{Phe}$, rrnS, $tRNA^{Val}$	0	MZ736693-MZ736708
2	12S[#]	F: AGACATAAAAACGTTAGGTC<br>R: GCTTTGGTTCAAAGTGGTCA	60	~350	rrnS, $tRNA^{Val}$	0	
3	P3[*]	F: GCACGCACACACCGCCCGTCAC<br>R: CGCTTTCTTAATTGRTGGCTGC	55	~600	rrnS, $tRNA^{Val}$, rrnL	0	
4	P4[*]	F: AGCCTGGTGATAGCTGGTTGTCC<br>R: AAGCTCCATAGGGTCTTCTCGTC	55	~650	rrnL	0	
5	P5[*]	F: CCGTGCAAAGGTAGCATAATC<br>R: CCTAGNACTTTTCGTTCNACT	55	~800	rrnL, $tRNA^{Leu}$, NADH1	0	
6	P6[*]	F: GTGGCAGAGCCCGGTAATTG<br>R: TTACTCTATCAAAGTAACTC	55	~950	NADH1, $tRNA^{Ile}$	0	
7	P7[*]	F: TYCGAGCATCHTAYCCHCGAT T<br>R: TGGTTTAGBCCBCCTCAKCCYCC	55	~800	NADH1, $tRNA^{Ile}$, $tRNA^{Gln}$, $tRNA^{Met}$, NADH2	0	
8	P8[*]	F: CACTTYTGAGTNCCAGAAGT<br>R: TAGGGTRTTTAGCTGTTAAC	55	~850	NADH2, $tRNA^{Trp}$, $tRNA^{Ala}$	0	
9	P9[*]	F: AGACCAAGAGCCTTCAAAGC<br>R: GCTTCWACTATDGADGATGC	55	~650	$tRNA^{Trp}$, $tRNA^{Ala}$, $tRNA^{Asn}$, $tRNA^{Cys}$, $tRNA^{Tyr}$, COX1	0	
10	P10[*]	F: GGNGGNTTYGGHAAYTGACT<br>R: GAARATRAAGCCTAGRGCTCA	55	~750	COX1	1	

S.No.	Primer	Sequence	Ta (°C)	Amplicon	Targeted genes	Polymorphic sites	Genbank accessions
11	P11*	F: TTYACHGTHGGAATAGAYGT<br>R: GCRTCTTGRAANCCTARTTG	55	~800	COX1, $tRNA^{Ser}$, $tRNA^{Asp}$, COX2	0	MZ736693-MZ736708
12	P12*	F: CCCCCYAYWRYTGGTTTCAAGCCA<br>R: GTKGAYGTRTCTAGTTGYGGCAT	55	~500	$tRNA^{Asp}$, COX2, $tRNA^{Lys}$, ATP8	0	
13	P13*	F: CAATGCTCHGARATYTGYGG<br>R: GANARDGCTCCYGTDAGNGGT	55	~950	COX2, $tRNA^{Lys}$, ATP8, ATP6, COX3	1	
14	P14*	F: GCCTAYGTNTTYACYCTNCTAGT<br>R: TGATTGGAAGTCARYTGTAC	55	~800	ATP6, COX3, $tRNA^{Gly}$, NADH3	1	
15	P16*	F: AGCYTGYGAAGCAGCACTAGG<br>R: GCKGTRGCTCCTATRTARCTTCA	55	~950	$tRNA^{Arg}$, NADH4L, NADH4	0	
16	P17*	F: AGCTCHATYTGYYTHCGYCAA AC<br>R: CCAATTTTTTGGYTCCTAAGRCC	55	~700	NADH4, tRNAHis, tRNASer, tRNALeu	1	
17	P18*	F: CCGAAAAAGYAYGCAAGAACTGC<br>R: GCDGATTTTCCDGTKGCDGCTA	55	~750	tRNASer, tRNALeu, NADH5	0	
18	P19*	F: GACGARCAGAYGCHAAYACAGC<br>R: GTDAKTADDAGGGCTCAGGCG	55	~700	NADH5	1	
19	P20*	F: GGMAGCCTNGCNYTAACAGG<br>R: AGTTTAATGGDHTDGGDGATTG	55	~600	NADH6	0	
20	P21*	F: CCATAACTRTAYAAAGCHGCAA<br>R: CCTCARAATGATATTTGKCCTCA	55	~650	NADH6, tRNAGlu, CYTB	3	
21	P22*	F: CAGGMCTATTCCTRGCHATAC	55	~950	CYTB, tRNAThr	1$21	

S.No.	Primer	Sequence	Ta (°C)	Amplicon	Targeted genes	Polymorphic sites	Genbank accessions
		R: CCCTTYTCTGGTTTACAAGACC					MZ736693-MZ736708
22	P23*	F: CATCGGACAACTAGCATCTAT	55	~650	tRNAThr, tRNAPro, CR	10	
		R: CCTGAAGWAAGAACCAGATG					
23	CRintF2#	F: GATTAATATTGCATAGTA	50	~500	CYTB, tRNA-Thr, tRNA-Pro,CR	5(3$23)	
	CRintR3#	R: AATAGTTAATAGAAAGGC					
24	CRF1#	F: CATGCCAGTATTAGTGAC	50	~450	tRNAPhe, CR	6$23	
	CRR1#	R: CAAGGTGTTGTTAGCTAC					
**Phylogeography Primers**							
25	P10*	F: GGNGGNTTYGGHAAYTGACT	45	431	COX1	1	MZ771364-MZ771458
	COXR#	R: GTAGACTTCAAAATGACC					
26	ATP6#	F: TCTAATACACTTAATCGG	50	379	ATP6, COX3	2	MZ771934- MZ772028
		R: CTTGGAATGTGCTTTCAC					
27	NADH4#	F: CCAAACACCATGAAGTTA	50	262	NADH4	1	MZ771459- MZ771553
		R: GATCATGAGAATGATGAC					
28	NADH5#	F: AACAAATCTTCACACTTG	50	268	NADH5	1	MZ771554- MZ771648
		R: ATAGGGTGGTGATTGCTC					
29	CYTB1#	F: AAATCTCACCCACTAGTT	50	428	CYTB	2	MZ771649- MZ771743
		R: TTTGTAATGACTGTAGCT					
30	CYTB2#	F: CTACACGAAACAGGGTCC	50	338	CYTB	1	MZ771744 - MZ771838
		R: ATTGGCTTAGGGGTCGGA					
31	CRF1#	F: CATGCCAGTATTAGTGAC	50	281	CR	7	MZ771839- MZ771933

S.No.	Primer	Sequence	Ta (°C)	Amplicon	Targeted genes	Polymorphic sites	Genbank accessions
	CRintR2#	R: ATACGCATGTTGACTGGA					MZ771839-MZ771933
32	CRintF2#	F: GATTAATATTGCATAGTA	50	495	CYTB, tRNA-Thr, tRNA-Pro,CR	6	
	CRintR3#	R: AATAGTTAATAGAAAGGC					

*Designed by Hassanin et al. 2011

[#] Designed in this study

[$] Overlapping number of segregating sites with the primer name

Additional file 1 Table S2: Sample (both tissue and dung) of Indian rhinos used in this study. A total of 111 individual rhino samples (72 tissue and 39 dung samples, respectively) were used in this study. Out of the 72 tissue samples, 16 samples representing different parks were used to generate the whole mitogenome data.

State	Protected areas	Total tissue samples[a]	Tissue used for whole mitogenome data[b]	Dung collected	Dung used for phylogeography[c]
Uttar Pradesh	Dudhwa National Park	4	2	13	6
West Bengal	Gorumara National Park	2	2	18	8
	Jaldapara National Park	4	2	15	6
Assam	Manas National Park	3	1	22	9
	Pobitora Wildlife Sanctuary	9	2	5	1
	Orang National Park	3	3	15	9
	Kaziranga National Park	46	3	0	0
Bihar*	Valmiki National Park	1	1	NA	NA
	**Total**	**72**	**16**	88	**39**

*Reference sample from non-rhino bearing area

[a] Total number of tissue samples used in this study. Out of 72, 16 were used for mitogenome data generation as population-wise details given in next column.

[b] Details of representative tissue samples to generate whole mitogenome data for identification of polymorphic sites across Indian rhino populations

[c] Dung details of identified rhino individuals representing six parks to ensure spatial coverage for phylogeography analysis. In total 111 samples were used for this work consisting of 72 tissues and 39 dungs.

Additional file 1 Table S3: Mitogenome organization in *Rhinoceros unicornis*. Codons respective to each tRNA are mentioned in parenthesis.

Genes	Position		Gene length	Spaces/ overlap	Strand	Codons	
	Start	Stop				Start	Stop
$tRNA^{Phe}$ (GAA)	1	69	69	0	H		
rrnS	70	1041	972	-1	H		
$tRNA^{Val}$ (TAC)	1041	1107	67	0	H		
rrnL	1108	2681	1574	3	H		
$tRNA^{Leu}$ (TAA)	2685	2759	75	2	H		
NADH1	2762	3718	957	-1	H	ATG	TAA
$tRNA^{Ile}$ (GAT)	3718	3786	69	-3	H		
$tRNA^{Gln}$ (TTG)	3784	3856	73	2	L		
$tRNA^{Met}$ (CAT)	3859	3927	69	0	H		
NADH2	3928	4971	1044	-2	H	ATA	TAG
$tRNA^{Trp}$ (TCA)	4970	5037	68	5	H		
$tRNA^{Ala}$ (TGC)	5043	5111	69	1	L		
$tRNA^{Asn}$ (GTT)	5113	5185	73	2	L		
OL	5188	5217	30	-1	H		
$tRNA^{Cys}$ (GCA)	5217	5282	66	0	L		
$tRNA^{Tyr}$ (GTA)	5283	5349	67	1	L		
COX1	5351	6895	1545	-3	H	ATG	TAA
$tRNA^{Ser}$ (TGA)	6893	6961	69	11	L		
$tRNA^{Asp}$ (GTC)	6973	7039	67	0	H		
COX2	7040	7723	684	3	H	ATG	TAA
$tRNA^{Lys}$ (TTT)	7727	7793	67	1	H		
ATP8	7795	7998	204	-43	H	ATG	TAA
ATP6	7956	8636	681	-1	H	ATG	TAA
COX3	8636	9420	785	-1	H	ATG	TAa
$tRNA^{Gly}$ (TCC)	9420	9488	69	-3	H		
NADH3	9486	9835	350	0	H	ATA	TAa
$tRNA^{Arg}$ (TCG)	9836	9905	70	0	H		
NADH4L	9906	10202	297	-7	H	ATG	TAA
NADH4	10196	11573	1378	0	H	ATG	Taa
$tRNA^{His}$ (GTG)	11574	11642	69	0	H		
$tRNA^{Ser}$ (GCT)	11643	11701	59	1	H		
$tRNA^{Leu}$ (TAG)	11703	11772	70	-9	H		
NADH5	11764	13593	1830	-17	H	ATA	TAA
NADH6	13577	14101	525	3	L	ATG	TAA
$tRNA^{Glu}$ (TTC)	14105	14173	69	4	L		
CYTB	14178	15317	1140	0	H	ATG	AGA
$tRNA^{Thr}$ (TGT)	15318	15386	69	1	H		
$tRNA^{Pro}$ (TGG)	15388	15453	66	268	L		
CR	15454	16828	1388	/	H		

Additional file 1 Table S4: Comparative analysis results of genetic diversity indices among five extant rhino species

Mitochondrial genes	African rhino			Sumatran rhino			Javan rhino			Indian rhino		
	**S**	**H_d**	**π**	**S**	**H_d**	**π**	**S**	**H_d**	**π**	**S**	**H_d**	**π**
**rrnS**	51	0.67	0.0352	0	0	0	1	0.53	0.0006	0	0	0
**rrnL**	68	0.67	0.0289	7	0.85	0.0014	13	0.73	0.0037	0	0	0
**ATP6**	64	0.67	0.0627	5	0.69	0.6857	4	0.80	0.8000	1	0.50	0.5000
**ATP8**	12	0.67	0.0392	1	0.42	0.0021	2	0.60	0.0043	0	0	0
**COX1**	100	0.67	0.0431	12	0.84	0.0033	11	0.93	0.0029	1	0.50	0.0003
**COX2**	49	0.67	0.0478	2	0.51	0.0008	4	0.73	0.7333	0	0	0
**COX3**	69	0.67	0.0587	3	0.54	0.0008	3	0.60	0.0018	1	0.13	0.0002
**CYTB**	98	0.67	0.0573	14	0.88	0.0050	8	0.73	0.0036	3	0.57	0.0011
**NADH1**	55	0.67	0.0384	6	0.78	0.0027	4	0.73	0.0020	0	0	0
**NADH2**	78	0.67	0.0499	7	0.89	0.0022	6	0.73	0.0029	0	0	0
**NADH3**	28	0.67	0.0540	5	0.81	0.0054	1	0.33	0.0010	0	0	0
**NADH4**	108	0.67	0.0523	14	0.73	0.0042	7	0.80	0.0023	1	0.33	0.0002
**NADH4L**	27	0.67	0.0606	2	0.26	0.0009	2	0.53	0.0036	0	0	0
**NADH5**	144	0.67	0.0527	14	0.81	0.0032	11	0.73	0.0031	1	0.50	0.0003
**NADH6**	51	0.67	0.0648	1	0.42	0.0008	4	0.73	0.0042	0	0	0
**tRNA**	65	0.67	0.0290	7	0.82	0.0016	0	0	0	1	0.33	0.0002
**CR**	185	0.67	0.1395	31	0.93	0.0108	21	0.93	0.0118	9	0.85	0.0045

Additional file 1 Table S5: Details of the variable sites based on concatenated sequence of 2531bp of Indian one horned rhino mtDNA haplotypes.

Clade	Gene		COX1	ATP6	COX3	NADH4	NADH5	CYTB			CR												
	Primers		P10F COXR	ATP6F ATP6R		NADH4F NADH4R	NADH5F NADH5R	CYTBF1 CYTBR1		CYTBF2 CYTBR2	CRF1 CRintR2					CRintF2 CRintR3							
	Position		5	8	8	1	1	1	1	1	1	1	1	1	1	1	1	1	1	1	1	1	1
			9	5	7	1	2	4	4	4	5	5	5	5	5	5	5	5	5	5	5	5	5
			7	2	9	2	4	2	5	8	3	5	6	6	6	7	7	7	7	8	8	8	8
			8	0	4	6	8	8	5	8	2	5	0	8	8	0	3	7	7	3	6	6	8
	Haplotype	N				0	9	5	5	2	2	9	6	5	7	3	0	0	6	5	4	8	4
Uttar Pradesh	H1	10	T	G	T	G	A	G	A	G	C	T	T	G	C	T	G	A	T	C	G	C	T
West Bengal	H2	20	*	*	*	A	*	A	G	A	T	*	C	*	T	*	A	*	*	*	*	T	C
Assam	H3	1	*	*	*	A	*	*	*	*	T	C	*	A	*	*	A	*	*	*	*	*	C
	H4	1	*	*	*	A	*	*	*	*	T	C	*	*	*	C	A	*	*	*	*	*	C
	H5	1	C	T	*	A	G	*	G	*	T	C	*	A	*	*	A	G	*	*	*	T	*
	H6	1	*	*	C	A	*	*	*	*	T	C	*	A	*	*	A	G	*	*	*	T	*
	H7	2	C	T	*	A	G	*	G	*	T	*	*	*	*	C	A	*	*	*	*	*	C
	H8	26	C	T	*	A	G	*	*	*	T	C	*	A	*	*	A	G	*	*	*	T	*
	H9	1	C	T	*	A	G	C	*	A	T	C	*	A	*	*	A	G	*	*	*	T	*
	H10	1	*	*	C	A	*	*	*	*	T	*	C	*	*	*	A	*	*	T	*	T	*
	H11	5	C	T	*	A	G	*	*	A	T	C	*	A	*	*	A	G	*	*	*	T	*
	H12	1	*	*	C	A	*	*	*	A	T	*	C	*	*	*	A	*	*	T	*	T	*
	H13	1	*	*	*	A	*	*	*	A	T	*	*	*	*	*	A	*	*	*	*	*	C
	H14	1	C	T	*	A	G	*	*	*	T	C	*	A	*	*	A	G	*	*	*	*	*
	H15	1	*	*	*	A	*	*	*	*	T	*	*	*	*	*	A	*	C	*	*	T	*
	H16	2	*	T	*	A	G	*	*	*	T	C	*	A	*	*	A	G	*	*	*	T	*
	H17	2	C	*	C	A	*	*	*	*	T	*	C	*	*	*	A	*	C	T	A	T	*
	H18	3	*	*	C	A	*	*	*	*	T	*	C	*	*	*	A	*	C	T	A	T	*
	H19	2	*	*	*	A	*	*	*	*	T	*	*	*	*	C	A	*	*	T	A	T	*
	H20	10	*	*	*	A	*	*	*	*	T	*	*	*	*	C	A	*	*	*	*	*	C
	H21	3	C	*	*	A	*	*	*	*	T	*	*	*	*	C	A	G	*	*	*	T	*
	H22	2	*	*	*	A	*	*	*	*	T	*	*	*	*	C	A	G	*	*	*	T	*
	H23	3	C	*	*	A	*	*	*	*	T	*	*	*	*	C	A	*	*	*	*	*	C
	H24	1	*	*	*	A	G	*	*	*	T	*	*	*	*	C	A	G	*	*	*	*	*
	H25	1	C	T	*	A	*	*	*	*	T	C	*	A	*	*	A	G	*	*	*	T	*
	H26	4	C	T	*	A	G	C	*	*	T	C	*	A	*	*	A	G	*	*	*	T	*
	H27	1	*	*	C	A	*	C	*	*	T	C	*	A	*	*	A	*	*	T	*	T	*
	H28	1	C	*	T	A	*	*	*	*	T	C	*	A	*	*	A	G	*	*	*	T	*
	H29	1	C	T	T	A	G	*	*	*	T	*	*	*	*	C	A	*	*	*	*	*	C
	H30	1	*	T	T	A	G	*	*	*	T	*	*	*	*	C	A	*	*	*	*	*	C

Positions of nucleotides corresponds to whole mitogenome of Indian one horned rhino

N number of samples

Red nucleotides represents the clade specific SNP
