# Supplementary material for: Consideration of genetic variation and evolutionary history in future conservation of Indian one-horned rhinoceros (Rhinoceros unicornis)
Source: BMC Ecol Evol. 2022 Jul 20;22:92. doi: 10.1186/s12862-022-02045-2 (PMC9301832; doi:10.1186/s12862-022-02045-2)

Additional file 2 legends

Additional file 2 Fig. S1: Whole mitogenome organisation and annotation of *Rhinoceros unicornis.*

Additional file 2 Fig. S2: Gene-wise comparative analyses of polymorphism indices (S, Hd and π) for all extant rhino species.

Additional file 2 Fig. S3: Estimation of mitogenome mutation rate for Indian rhino using Caballine (Zebra, Donkey, Asiatic Wild Ass and Horse) as outgroup. Internal node calibration points are presented in italics (bold). Estimated node ages and branch mutation rates with available reference in literature is marked with * (Fernando et al., 2006; Steiner et al., 2011; Steiner et al., 2017; Liu et al., 2021).

Additional file 2 Fig. S1

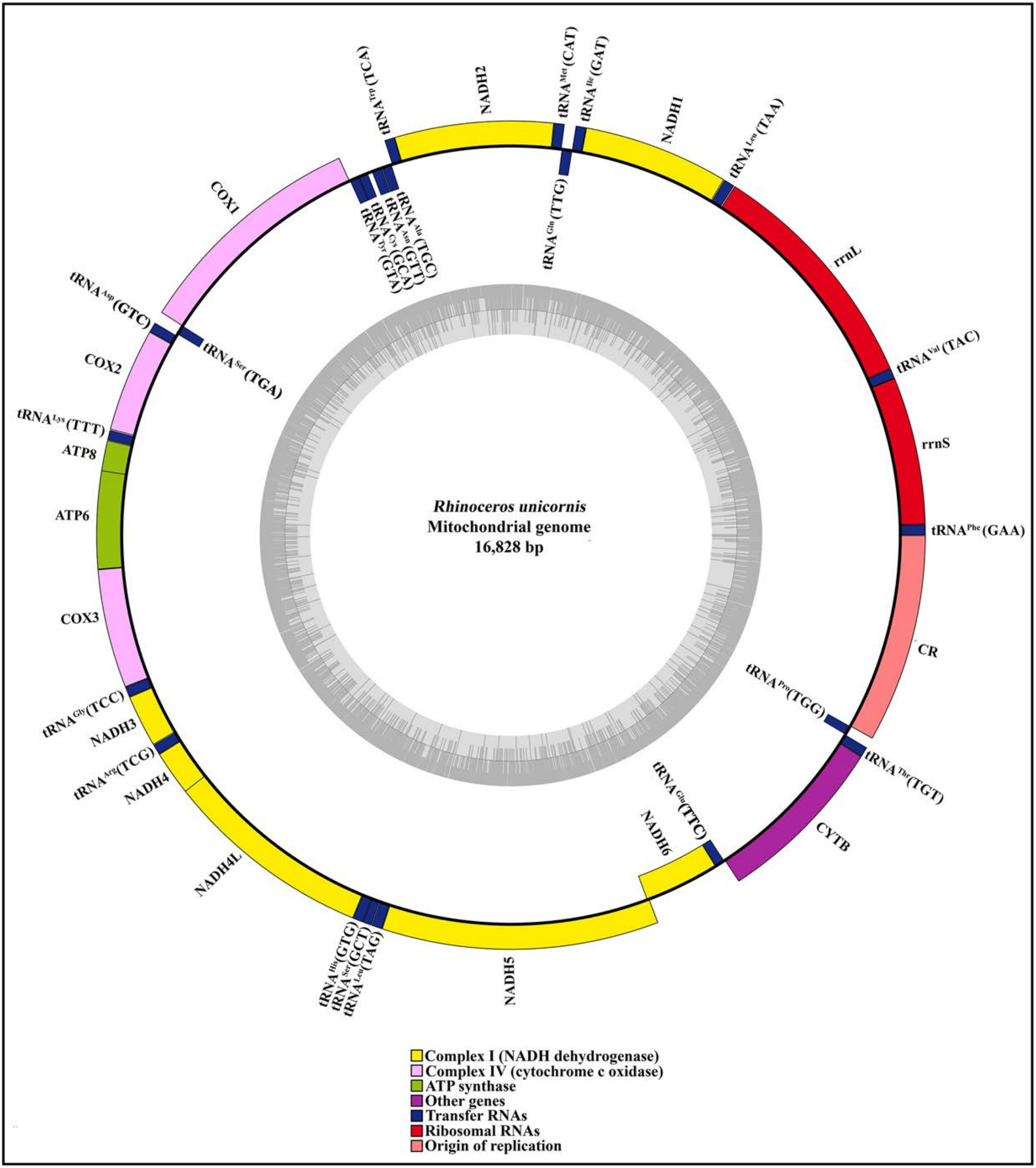

Additional file 2 Fig. S2

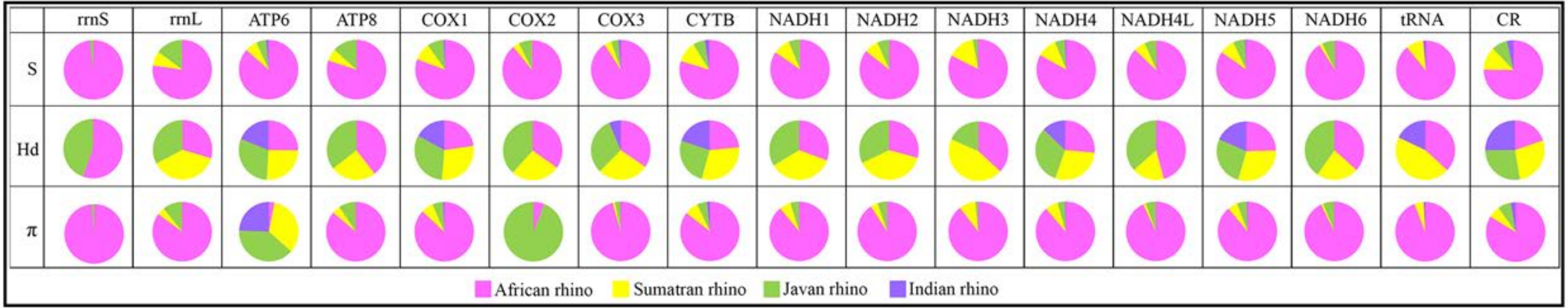

Additional file 2 Fig. S3

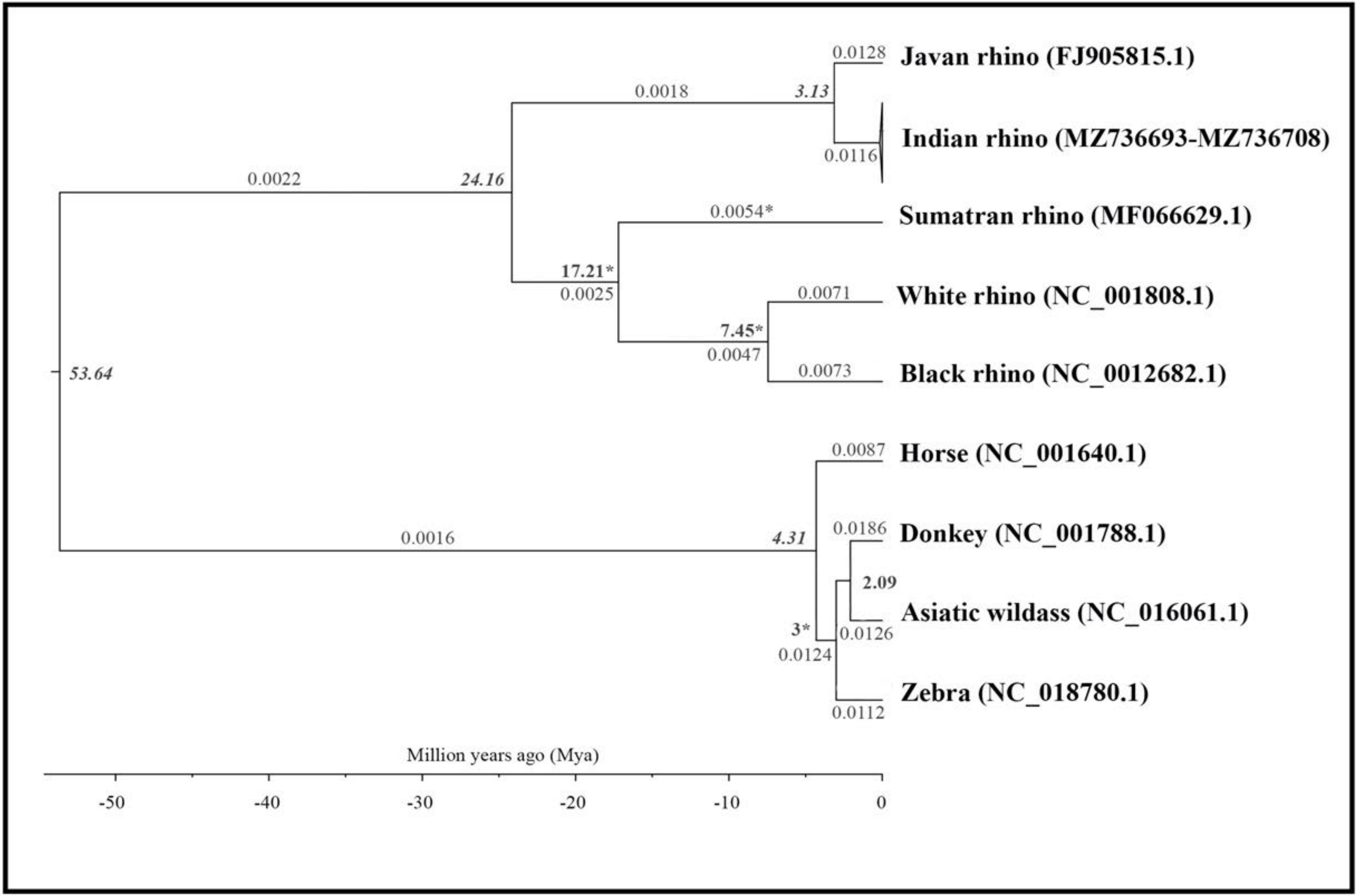

Supplement: Supplementary file 2 — Additional file 2: Figure S1. Whole mitogenome organisation and annotation of Rhinoceros unicornis. Figure S2. Gene-wise comparative analyses of polymorphism indices (S, Hd and π) for all extant rhino species. Figure S3. Estimation of mitogenome mutation rate for Indian rhino using Caballine (Zebra, Donkey, Asiatic Wild Ass and Horse) as outgroup. Internal node calibration points are presented in italics (bold). Estimated node ages and branch mutation rates with available reference in literature is marked with * [1, 8, 10, 29]. [file 12862_2022_2045_MOESM2_ESM.pdf]
